# Dietary Restriction of Advanced Glycation End-Products (AGEs) in Patients with Diabetes: A Systematic Review of Randomized Controlled Trials

**DOI:** 10.3390/ijms252111407

**Published:** 2024-10-23

**Authors:** Paraskevi Detopoulou, Gavriela Voulgaridou, Vasiliki Seva, Odysseas Kounetakis, Ios-Ioanna Desli, Despoina Tsoumana, Vasilios Dedes, Evridiki Papachristou, Sousana Papadopoulou, George Panoutsopoulos

**Affiliations:** 1 Department of Nutritional Sciences and Dietetics, University of the Peloponnese, 24100 Kalamata, Greece; 2 Department of Clinical Nutrition, General Hospital Korgialenio Benakio, 11526 Athens, Greece; 3 Department of Nutritional Sciences and Dietetics, School of Health Sciences, International Hellenic University, 57400 Thessaloniki, Greece

**Keywords:** advanced glycation end-products, diet, diabetes, dietary intervention

## Abstract

Advanced Glycation End Products (AGEs) are formed through non-enzymatic reactions between reducing sugars and proteins, nucleic acids or lipids (for example through hyperoxidation). In diabetes, elevated glucose levels provide more substrate for AGEs formation. AGEs can also be ingested through the diet from foods cooked at high temperatures, or containing much sugar. The present work aimed to review all published randomized controlled trials (RCT) on low-dietary AGE (L-dAGEs) interventions in patients with diabetes. Pubmed, Scopus and Cochrane databases were searched (until 29 February 2024) with appropriate keywords (inclusion criteria: RCT, patients with diabetes, age > 18 years, outcomes related to inflammation, glucose, and lipids; exclusion criteria: non-RCTs, case-series, case reports and Letter to the Editor, or animal studies). The present review was registered to the Open Science Framework (OSF). From 7091 studies, seven were ultimately included. Bias was assessed with the updated Cochrane Risk of Bias tool. A reduction in circulating AGEs was documented in 3/3 studies. No particular differences were documented in glycemic parameters after a L-dAGEs diet. Reductions in glucose levels were observed in one out of six studies (1/6), while HbA1c and HOMA did not change in any study (0/6 and 0/3, correspondingly). Lipid profile also changed in one out of four studies (1/4). More consistent results were observed for oxidative stress (beneficial effects in 3/3 studies) and inflammatory markers (beneficial effects in 4/4 studies). Other athero-protective effects, such as adiponectin increases, were reported. Limitations included the small sample size and the fact that dietary and physical activity habits were not considered in most studies. In conclusion, a L-dAGEs pattern may minimize AGEs accumulation and have beneficial effects on oxidative stress and inflammation indices, while its effects on glycemic and lipemic parameters are inconsistent and modest in patients with diabetes.

## 1. Introduction

Advanced Glycation End Products (AGEs) are a group of complex molecules formed through non-enzymatic reactions between reducing sugars and proteins, or nucleic acids [1]. Lipids can also lead to AGE formation [2,3]. In increased oxidative stress, the oxidation of fatty acids and glucose can lead to the formation of reactive carbonyls, such as methylglyoxal, which can further produce AGEs [4]. For example, Ne-carboxymethyl-lysine (CML) can be formed by several nonenzymatic glycation and oxidation reactions, but also by oxidation of polyunsaturated fatty acids in the presence of protein [5].

**Figure 1:**
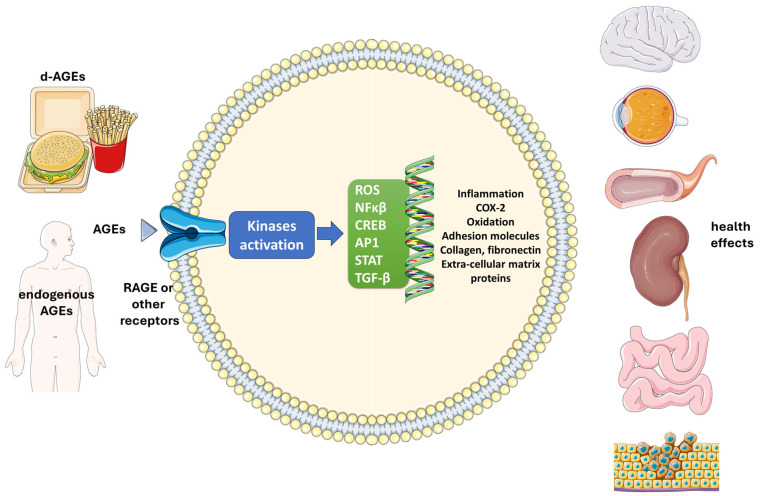
Mechanistic aspects of AGEs. Dietary AGEs or AGEs produced in the body (endogenous) exert their effects through binding to specific receptors, such as RAGE (multiligand pattern recognition receptor) or other receptors, such as stabilin-1 and stabilin-2. Then, several kinases and signaling pathways are activated, leading to the generation of reactive oxygen species (ROS), nuclear-factor κβ, and other molecules that regulate gene expression. In turn, cellular responses lead to inflammation, oxidative stress and activation/increases of COX-2, adhesion molecules, collagen, fibronectin and extra-cellular matrix proteins. As a result, systemic effects may be observed in several organs and tissues (brain, eyes, kidney, intestine, vessels), while diabetes complications and/or other diseases may be triggered, such as cancer. Icons were taken from Servier Medical Art, licensed under CC BY 4.0 (https://smart.servier.com/). AGE: Advanced-glycation end products; d-AGEs: dietary advanced-glycation end products; ROS: Reactive oxygen species; NFkβ: nuclear-factor-kβ; CREB: cAMP-response element binding protein; AP1: activator protein 1; STAT: signal transducer and activator of transcription; TGF-β: transforming growth factor beta; COX-2: cyclooxygenase-2.

AGEs are formed through the non-enzymatic reaction between reducing sugars and amino groups in proteins, lipids, or nucleic acids. This process, known as glycation, leads to the formation of stable, covalent crosslinks between proteins, which can significantly alter their structure and function [6]. They also exert their effects through interaction with specific receptors, such as RAGE (multiligand pattern recognition receptor), which are expressed on various cell types, including endothelial cells, smooth muscle cells, macrophages, and neurons [7]. AGE accumulation can activate RAGE receptors, leading to the increased generation of reactive oxygen species and also activates signaling pathways that lead to inflammation [8]. This activation initiates downstream signaling pathways that regulate gene expression, influencing various cellular responses [9]. Indeed, AGEs binding to RAGE triggers intracellular signaling pathways, including nuclear factor-kappa B (NF-κB) and mitogen-activated protein (MAP) kinases [7]. These pathways lead to the production of pro-inflammatory cytokines, adhesion molecules, and reactive oxygen species (ROS) [7]. The AGE–RAGE interaction plays a crucial role in mediating the biological effects associated with the accumulation of AGEs in tissues. Indeed, AGEs and AGE–RAGE interactions are implicated in several age-related diseases and conditions, including diabetes, atherosclerosis, Alzheimer’s disease, chronic kidney disease, and cataracts [1]. It is noted that AGEs can bind to several other receptors, such as AGE-R1, AGE-R2, and AGE-R3 receptors, as well as to scavenger receptors, such as stabilin-1 and stabilin-2. Ligands other than AGEs can also bind to all these receptors [10] (Figure 1).

In diabetes, elevated blood glucose levels provide more substrate for the formation of AGEs through the glycation process [6]. AGEs can then contribute to tissue damage through proteins modification [6]. For example, AGE accumulation in retinal vessels relates to the severity of retinopathy [11], while protein modification in blood vessels leads to endothelial dysfunction, increased permeability, and atherosclerosis [6]. AGEs also contribute to other complications of diabetes, such as the development and progression of diabetic nephropathy and neuropathy [6]. In parallel, the presence of AGEs triggers oxidative stress and inflammatory responses, promoting chronic inflammation in the body [8]. Chronic inflammation and oxidative stress may, in turn, further damage pancreatic beta cells and cause insulin resistance, causing increases in blood glucose and glycation [12]. In this context, the control of circulating AGEs may have an effect on the course of the disease.

AGEs can be endogenously formed or ingested through the diet, particularly from foods that are cooked at high temperatures, or that contain high levels of sugars. For example, cooking methods like grilling, frying, or roasting can promote the formation of AGEs in foods. In addition, ultra-processed foods may have a high AGE content [13], since they are usually cooked or heated at high temperatures to ensure food safety and enhance their characteristics, such as flavor and color [13]. Dietary AGEs are absorbed at a rate of 10–30% [14]. The AGEs escaping digestion and absorption go to the colon, where they may modify gut microbiome metabolism and short-chain fatty acid production [15]. Despite their relatively low absorption rate, dietary AGEs may lead to increased glycation reactions, and contribute significantly to the body’s AGEs pool [16,17].

Previous systematic reviews and meta-analyses support the view that the adoption of low-AGE diets improves the lipid profile and insulin resistance [18], while differentiations may exist according to health status [19]. In addition, low-AGE diets have been connected to reductions in inflammatory markers, 8-isoprostane, leptin, circulating AGE levels, RAGE, as well as to increased adiponectin levels and mononuclear cells sirtuin-1 in healthy subjects and patients with diabetes [19]. Previous work has searched related articles up to May 2016 [19], while the meta-analysis of Sohouli was published in 2021 and includes more recent studies [18]. However, the work of Sohouli et al. examines the effects of low-AGE diets in healthy subjects and patients with diabetes, obesity and other diseases (grouped analysis) [18], while differentiations may exist in patients with diabetes [19].

Therefore, the aim of the present work was to review all published randomized controlled trials concerning the effects of a low-AGE diet in patients with diabetes by also including all recent studies. Moreover, no limitations were applied to the investigated outcomes.

## 2. Materials and Methods

This systematic review was performed according to PRISMA guidelines [20] (Supplementary Files S1 and S2). The present review was registered to Open Science Framework (OSF) (osf.io/4dqkr; https://doi.org/10.17605/OSF.IO/GNVER, accessed on 18 October 2024). A protocol for this review has not been published.

### 2.1. Search Strategy

Three literature databases (PubMed, Scopus, Cochrane) were searched using the terms “Advanced glycation end-product”, “Diabetes Mellitus”, “inflammation”, “glycemic profile” and “lipid profile” to identify relevant articles. Synonymous words for these terms were combined using the Boolean operator “OR”, and the terms were further combined using the Boolean operator “AND”. The search query applied in PubMed is detailed in Table 1. The research question was formulated as follows: “Did patients with diabetes who followed a restricted AGEs diet experience changes in glycemic, lipid, and inflammatory profiles compared to those following a standard diet?”. The literature was searched until the end of February 2024.

### 2.2. Primary and Secondary Outcomes

The primary outcomes assessed in this study were the changes in glycemic parameters (including fasting glucose, HbA1c and HOMA) and lipid profile (such as triglycerides, LDL cholesterol and HDL cholesterol) among patients with diabetes following the intervention. Additionally, changes in inflammatory markers, as defined by markers like CRP and TNF-α, were also considered primary outcomes. Secondary outcomes included markers associated with cardiometabolic risk and kidney function. Moreover, the study incorporated the analysis of changes in specific circulating serum AGE levels following the intervention. It is noted that the mean differences are not presented, since they were not uniformly reported by all studies.

### 2.3. Inclusion and Exclusion Criteria

Studies were eligible for the systematic review if they were randomized controlled trials, participants were type 2 diabetes patients > 18 years of age and the outcomes were related to inflammation, glucose levels and lipidemic profile. Exclusion criteria were: (1) non-RCTs, (2) case-series, case reports and Letter to the Editor, (3) animal studies.

### 2.4. Quality Assessment

The updated Cochrane Risk of Bias tool (RoB2) [21] was utilized to evaluate the risk of bias in the included studies. This tool assesses five parameters: random sampling, intervention methodology, missing data, outcome assessment and presentation of results. Two researchers (VS and OK) independently assessed the studies for risk of bias and categorized them as low, moderate, or high risk of bias based on the RoB2 results. In case of any disagreement between the two researchers, a third, more experienced researcher intervened to resolve it (GV).

### 2.5. Data Selection Process and Data Extraction

Two independent researchers initially screened all retrieved articles (VS, OK) based on their titles and abstracts, excluding those that did not meet the inclusion criteria. Subsequently, the remaining articles were screened by full text to identify those meeting the inclusion criteria. The results of the included studies were then extracted into a predefined Excel spreadsheet. The extracted data included details of the studies (such as first author, year, journal, masking and randomization), characteristics of the study population (total number, age of participants, diabetes mellitus diagnosis, inclusion and exclusion criteria), characteristics of the intervention and control groups, study duration, primary and secondary outcomes, and results. Any disagreements between the researchers during the data selection and extraction processes were resolved through discussion with a third researcher (GV).

## 3. Results

**Figure 2:**
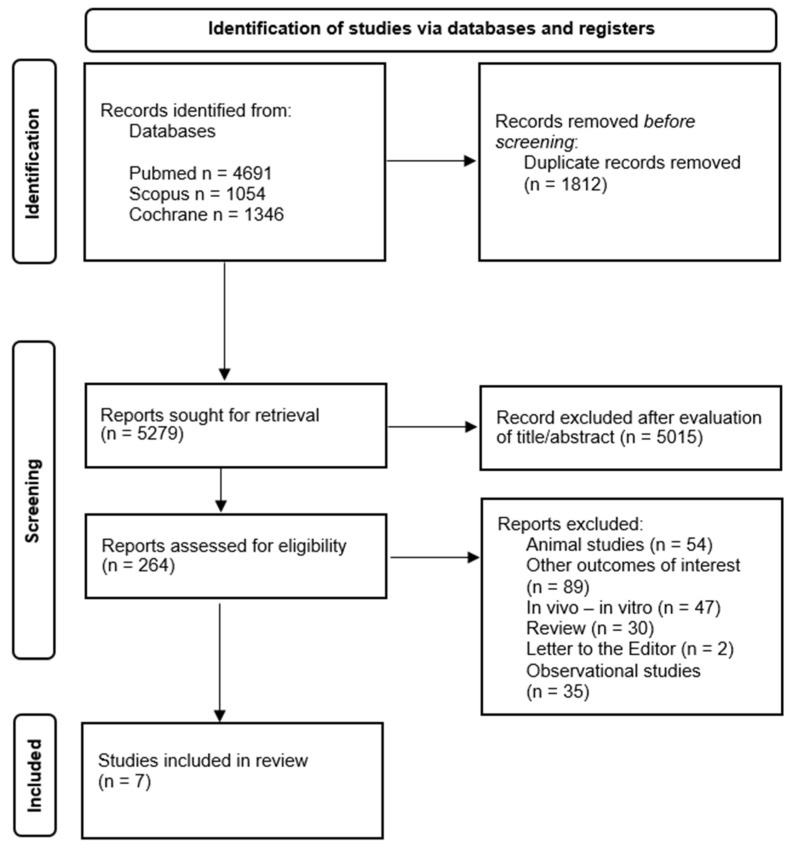
Flow diagram of the screening process.

From a total of 7091 search results, seven met the inclusion criteria and were included in the final systematic review. Figure 2 presents the selection process of the retrieved studies from the three literature databases.

### 3.1. Study Design

Seven randomized controlled trials including 263 patients with Diabetes Mellitus were identified [22,23,24,25,26,27,28]. The studies were conducted in Italy [23], Israel [24], Belgium [26], Mexico [25] and the USA [22,28]. The majority of the studies included both males and females [22,24,25,26,27,28], and only one study did not mention participants’ sex [23]. The mean age of the participants ranged from 46 to 73.9 years. (Table 2 and Table 3).

### 3.2. Low-AGE Intervention

All the included studies had a group that followed a diet low in dietary AGEs (L-dAGEs) and a control group that was advised to follow their standard nutrition (S-dAGEs) (Table 4). Key features of the L-dAGEs diet intervention were written information regarding

(i) cooking methods (avoid high-heat, dry cooking methods like frying, roasting, baking and grilling, and choose low-temperature, moist-heat methods like boiling, steaming, poaching, or stewing);
(ii) time of cooking;
(iii) food selection: focus on unprocessed, fresh foods, particularly low-fat or lean meats, and limit processed foods high in AGEs, such as processed meats, full-fat cheeses and baked goods.

The interventions were similar in terms of provided energy [22,23,25,27,28] and macronutrients [22,23,25,28], except one study that found lower energy and macronutrients intake in the L-dAGEs group [24]. In one study these variables were not reported [26].

### 3.3. Biomarkers Assessed

Glucose [22,23,24,25,27,28], HbA1c [22,23,24,25,26,27,28] and insulin HOMA-IR [24] were measured to estimate the glycemic profile. Total cholesterol, triglycerides, HDL-cholesterol and LDL-cholesterol were measured as indicators for lipid profile [22,23,24,25,28], whereas, in the study of Di Pino et al., ApoA and ApoB were also measured [23]. The inflammatory profile was estimated by circulating hs-CRP and TNF-α [22,23,25,28]. One study also assessed sirtuin expression [27] and adiponectin levels [27].

Oxidative stress was evaluated in three studies by estimating 8-isoprostanes [27], malondialdehyde (MDA) [25] and oxidized LDL [22]. Several serum AGEs and receptors of AGEs were measured only in three studies: CML, AGE receptor-1 (AGER1) [27], Ne-carboxyethyl lysine (CML), 3-deoxyglucosone hydroimidazolone (3DG-H1) [24] and methylglyoxal (MG) [24,27,28]. Some studies also evaluated kidney function through eGFR [26], urea and creatinine [24].

### 3.4. Results of Low-AGE Diets on Glycemic Profile

No particular differences were documented in glycemic parameters after a L-dAGEs diet. Reductions in glucose levels were observed in one [28] out of six studies [22,23,24,25,27,28] (1/6). Similarly, HbA1c did not change in any study (0/6) [22,23,24,25,26,28], and HOMA did not change in any study (0/3) [24,25,27]. In one study, both the S-dAGEs and L-dAGEs groups showed a decrease in insulin and HOMA-IR, with no significant difference between them [24], while insulin levels did not change in the control and intervention groups (Table 3) [25].

### 3.5. Results of Low-AGE Diets on Lipid Profile

Total cholesterol, LDL-cholesterol and non-HDL-cholesterol were reduced in the L-dAGEs group compared with the S-dAGEs groups in 12 and 24 weeks [22,23], while after 6 weeks no difference was documented [22,25,28] (beneficial difference in one out of four studies; 1/4). Similarly, there was a reduction in triglycerides and non-HDL cholesterol in the L-dAGEs group compared to the S-dAGEs group only after 12 weeks of the intervention [23]. In the same study, a reduction of ApoB and ApoB/ApoA ratio was observed in the L-dAGEs group from baseline and compared to S-dAGEs after 24 weeks of the intervention [23]. Interestingly, LDL from diabetic patients on L-dAGEs diet was less glycated (by 50%) and less oxidized vs. a S-dAGEs diet [22] (Table 3).

### 3.6. Results of Low-AGE Diets on Serum AGEs

Circulating AGEs decreased following L-dAGEs interventions in all studies (3/3). More particularly, in one study, an increase in circulating AGE (CEL and MG-H1) concentrations was observed in the S-dAGEs group compared to the L-dAGEs group [24]. In an intention-to-treat (ITT) analysis, a significant decrease in CEL, 3DGH and MG-H1 serum AGEs was observed in participants classified as “very highly adherent” compared to those classified as “less adherent in the L-dAGEs diet group” [24]. Furthermore, in patients with diabetes following an L-dAGEs diet for four months, there was a decrease in sCML, sMG (serum methyl-glyoxal) and iMG (intracellular methyl-glyoxal) levels, while in those following a S-dAGEs diet, sMG and iCML levels increased. Significant changes over time were observed between the two groups in sCML, sMG, iCML and iMG markers [27]. In contrast, AGER1 levels in peripheral blood mononuclear cells increased in patients following an L-dAGEs diet [27]. Additionally, in healthy patients who received the L-dAGEs diet, sCML and sMG levels decreased significantly compared to those in the S-dAGEs group [27] (Table 3). Serum CML was also reduced in the study of Vlassara et al. by 40% [28].

### 3.7. Results of Low-AGE Diets on Inflammatory Profile

Markers of inflammation decreased following L-dAGEs interventions in all studies that were measured (4/4) [22,23,25,28]. Most studies revealed reductions in inflammatory markers CRP [22,28] and mononuclear TNA-a [22,28] or serum TNF-a [25]. In another study, reduced hs-CRP levels were observed only after 24 weeks of intervention in the L-dAGEs group with no difference, versus the S-dAGEs group [23], while in another study no difference in CRP was documented [25].

### 3.8. Results of Low-AGE Diets on Other Parameters

Several lines of evidence suggest athero-protective effects of the L-dAGEs diet. A significant decrease in intima-media thickness (IMT) in the L-dAGEs after 24 weeks of the intervention was observed compared to the baseline [23]. Additionally, an increase in adiponectin and a decrease in leptin was observed after four months of intervention in patients with diabetes following an L-dAGEs diet compared to a standard S-AGE diet [27]. Markers of oxidative stress tended to decrease after L-dAGEs diets. More specifically, 8-isoprostanes levels decreased in the L-dAGEs group in both patients with diabetes and healthy participants [27], while a decrease in MDA was also documented [25]. SIRT-1 levels increased in patients with diabetes following an L-dAGEs diet [27].

Regarding renal function, eGFR did not change during the intervention in the group following the L-AGE diet, or between the L-AGE group and the S-AGE group [26].

### 3.9. Risk of Bias Assessment

Table 5 summarizes the risk of bias of the included studies. Five studies were rated with an unclear risk in random sequence generation [22,24,26,27,28], and two studies were rated with an unclear risk in incomplete outcome data [24,25]. All included studies were rated with an unclear risk in allocation concealment, blinding of both participants and personnel, blinding of outcomes assessment, and other bias [22,23,24,25,26,27,28]. Four studies were rated as having an unclear risk of bias in selective reporting [22,25,26,28] and the other three studies [23,24,27] were rated as having a low risk of bias. All included studies were assessed as having an unclear risk of bias, as we found that they had more than one domain of concern.

## 4. Discussion

The present systematic review included seven randomized controlled trials that assessed the effects of a L-dAGEs diet in patients with diabetes on multiple outcomes, i.e., glycemic profile, lipemic profile, inflammatory markers, circulating AGEs, markers related to atherosclerosis and renal function. No particular differences were documented in glycemic parameters after a L-dAGEs diet. Reductions in glucose levels were observed in one out of six studies (1/6), while HbA1c and HOMA did not change in any study (0/6 and 0/3, correspondingly). Lipid profile also changed in one out of four studies (1/4). More consistent results were observed for oxidative stress (beneficial effects in 3/3 studies) and inflammatory markers (beneficial effects in 4/4 studies). In parallel, one study documented favorable effects in the leptin/adiponectin ratio, suggesting potential athero-protective effects of a L-dAGEs diet (1/1 study).

No particular differences were documented in glycemic parameters after a L-dAGEs diet. The relationship of AGEs to insulin sensitivity has been previously reported [19,29], while an excessive ingestion of dietary AGEs promotes insulin resistance in mice [30]. Indeed, AGEs can increase insulin resistance through their interaction with RAGEs [31]. RAGEs increase the production of reactive oxygen species in adipocytes [31] and relate to adipocyte hypertrophy, suppression of glucose transporter type 4 and adiponectin expression [32]. In addition, the consumption of ultra-processed foods (high in AGEs) has been connected to low adherence to healthy dietary patterns [33,34], which may also explain their inverse relation with insulin sensitivity. It is noted that the length of follow-up and the presence of diabetes may affect glucose levels, suggesting that a follow-up of more than eight weeks may be needed to detect potential differences [18]. Moreover, all but one study [25] included patients with good baseline values of glucose or HbA1c, which may be a reason why most studies did not find differences in glycemic profile after a low d-AGEs diet.

The effects of low d-AGEs were inconsistent for lipids, since only one study induced beneficial changes in the lipid profile of subjects with diabetes [23]. Such an effect has been previously reported [29]. Similarly, according to a meta-analysis [19], a low dietary consumption of AGEs was related to total cholesterol reduction in individuals without diabetes. The results of the present systematic review suggest a possibility of the positive effects of a low d-AGEs regimen also for persons with diabetes, although existing data are not very encouraging.

All studies that measured inflammatory and/or oxidative stress markers showed positive results. In subjects with type 2 diabetes, a diet high in AGEs increased inflammatory markers, i.e., C-Reactive protein, TNF-α, VCAM-1 [28] and oxidative stress. Indeed, by binding to several receptors, AGEs activate NADPH oxidase, leading to reactive oxygen species (ROS) production. In parallel, AGEs’ effects seem to also relate to platelet activating factor (PAF) levels [35], which, in turn, are implicated in cardiovascular disease [36] and metabolic abnormalities [37]. Interestingly, a diet rich in antioxidants counterbalancing the deleterious effects of AGEs has been shown to modulate PAF levels [38]. In parallel, AGE restriction (by 50%) lowered circulating AGEs and markers of oxidative stress in patients with diabetes [27]. Recent evidence also shows that a L-dAGEs diet can reduce COX-2 expression in monocytes in patients undergoing hemodialysis, reflecting the potential of a L-dAGEs diet to modify inflammation in a highly inflammatory milieu [39].

The degree of dAGEs restriction required to achieve health benefits is difficult to define. As mentioned, some studies have applied a 44% [25] to 50% dAGES reduction [27], while others have tested a much higher restriction (five-fold fewer AGEs) [22,23,28]. According to research, the mean consumption of d-AGEs is 16,000 AGEs −20,000 kU/day [40,41], which means that at least half of this quantity should be ingested to have some benefits. One study suggested much smaller differences pre-intervention [14.2 (11.3–19.4) MU AGEs/day] and post- intervention [11.6 (9.4–13.0) MU AGEs/day] [26], while others did not report the exact magnitude of AGE restriction, but provided only qualitative characteristics of the intervention [23,24]. It is noteworthy that the particular study documenting low changes in the dAGEs content also failed to document the clinical effects of the provided diet (no changes in HbA1c, and no changes in urine AGE-associated fluorescence) [26].

Several foods contain AGEs. The highest dietary CML levels from ELISA-derived measurements have been observed in beef and cheese, followed by poultry, pork and fish [13]. Roasted nuts contain 6447–9807 kU CML/100 g. However, the AGE content of foods may differ significantly according to cooking methods. CML and CEL (detected by UPLC-MS/MS) almost doubled when the cooking temperature of sausages changed from 70–90 °C to 130 °C [42]. Breakfast cereals also constitute a significant dietary source of AGEs [43]. Of note, the ELISA databases on AGE content in foods may have flaws [44,45,46] versus databases with chemically defined AGEs by LC/MS-MS (mg/100 g), and ELISA may overestimate the AGE content of fatty foods [43]. Although the absolute quantification of dAGEs may be difficult, the suggested interventions to lower AGE intake are still meant to contain low dAGEs.

The absorption of dAGEs is not yet fully understood [15]. It is also noted that low molecular AGEs from dietary sources are mostly absorbed into the circulation [15]. The non-absorbed AGEs proceed to the gastrointestinal tract, where they can modify local microbiota and gut health, with possible unfavorable effects [47].

Regarding the association of dAGEs and circulating AGEs, it has been previously shown that dAGEs can modulate circulating AGEs in healthy subjects [48], overweight subjects [49], and subjects with metabolic syndrome [50], diabetes [24,27] and renal problems [51,52]. In the present systematic review, two studies assessing circulating AGEs [24,27], and one study including urine AGEs-associated fluorescence, were included [26]. Lotan et al. assessed serum AGE concentrations by mass spectrometry [24] in contrast to the study of Uribarri et al. that used ELISA [27]. At this point, it should be stressed that ELISA methods capture the levels of plasma protein-bound AGEs. It was noted in the study of Steenbeke that dAGEs did not correspond with urine AGEs-associated fluorescence [26], but this study included elderly subjects with diabetic nephropathy, in whom AGEs metabolism may be differentiated [26]. Indeed, it should be acknowledged that circulating AGEs are also influenced by other factors except diet, such as age, glucose levels, protein metabolism, oxidative stress, inflammation, smoking, liver status and kidney status [53]. In this context, it is possible that L-dAGEs diets also modify oxidative stress levels, and thus indirectly all forms of circulating AGEs (free and protein-bound ones). Of note, the kidney plays a main role in the elimination of circulating low-molecular AGEs. In other words, the AGEs quantity in the body is determined by a balance reflecting their production and elimination rate by endogenous enzymatic systems [54].

L-dAGEs diets may also be low in other molecules. More particularly, L-dAGEs diets are commonly considered as diets containing minimally processed foods and foods cooked in low temperatures. However, since heat treatment also increases other substances, such as a-dicarbonyls (AGE precursors), acrylamide, 5-hydroxymethylfurfural and heterocyclic aromatic amines, L-dAGEs are also low in these molecules. The presented results may partially be due to the parallel reductions in these molecules [55]. In addition, studies do not document whether the L-dAGEs diets have similar energy and fat content to S-dAGEs diets, while higher cooking temperatures lead to higher destruction of thermo-labile essential nutrients [56]. Thus, the outcomes measured may also be attributed to other differences except dAGEs content. Moreover, the dAGEs load between S-dAGEs and L-dAGEs diets is not given in all studies.

When reviewing the age, obesity and progression of diabetes in the included studies, it is clear that the results may not be fully generalizable due to the characteristics of the participants. The studies reviewed mainly included older adults with type 2 diabetes, aged around 61 to 74 years [22,24,26,27,28], with the exception of those by Luévano-Contreras et al. [25] and di Pino et al. [23], who included younger subjects. Moreover, participants had a high BMI in all studies (mean >28 kg/m^2^) [22,23,24,25,26,27,28] and had good control of diabetes (HbA1c~6.7% to 7.3% or fasting glucose ~120 mg/dL) in all [22,23,24,26,27,28] but one study [25]. Older, overweight adults may already have a higher accumulation of AGEs due to natural aging and the inflammatory milieu induced by obesity. In addition, since participants already had relatively controlled diabetes, the observable effects of the L d-AGEs intervention on glucose levels or other metabolic markers may be modest. Therefore, the results of the present review may be best applied to older adults with type 2 diabetes, who are overweight or obese and have relatively well-controlled blood sugar levels. In contrast, the present results may not fully apply to younger, normal-weight individuals with early-stage diabetes or prediabetes.

A limitation of the reviewed studies is the small sample size, which may also affect the generalizability of the results. In addition, there is no uniform dietary regimen to limit AGEs, while L-dAGEs diets are also low in other substances, as mentioned above. Moreover, the confounding role of weight changes in the observed results cannot be excluded [23]. It is noteworthy that weight loss has been reported to lower circulating AGEs [57]. Furthermore, other confounding factors, known to modulate oxidative stress and inflammation, such as dietary and physical activity habits, were not considered in most studies. Last but not least, the risk of bias in the retained studies was rather unclear.

## 5. Conclusions

The present work showcases the beneficial effects of a L-dAGEs intervention in inflammatory and oxidative stress markers in patients with diabetes, while changes in glucose and lipids are inconsistent. A total elimination of foods high in AGEs (such as cheese and meat) could pose some risks such as micronutrient or vitamin deficiencies in the long-term, or it could connect to low protein intake. L-dAGEs interventions in subjects with diabetes have shown reductions of energy and macronutrient intake in the elderly [24] or no changes in younger subjects ~45 y [25] and 60 y [22]. However, adopting gentler cooking methods like steaming, slow cooking, or poaching can lower AGE content without sacrificing nutritional variety and macronutrient intake. Future research on dAGEs and diabetes should focus on clarifying the effects of d-AGEs on glycemic and lipemic profiles in a range of age-groups and both controlled and non-controlled subjects. Moreover, metabolic pathways of AGE absorption and their effects on the microbiome need to be studied. Long-term studies on the benefits of L-dAGE diets are also needed to assess their impact on reducing diabetic complications.

## Figures and Tables

**Table 1:** Search strategy in Pubmed.

#1 “Glycation End Products, Advanced” [Mesh] OR “Receptor for Advanced Glycation End Products” [Mesh] OR “Advanced glycation end-product” [tiab] OR “advanced glycation end product-receptor” [tiab]
#2 “diabetes mellitus” [tiab] OR “Diabetes Mellitus” [Mesh] OR “Diabetes Mellitus, Type 2” [Mesh] OR prediabetes [tiab] OR “Prediabetic State” [Mesh] OR “Metabolic Syndrome” [Mesh] OR “Metabolic Syndrome” [tiab]
#3 glucose [tiab] OR “glycemic profile” [tiab] OR “glucose levels” [tiab] OR “C-Reactive Protein” [Mesh] OR CRP [tiab] OR “C-Reactive Protein” [tiab] OR “inflammatory profile” [tiab] OR “inflammation” [tiab] OR “inflammatory indexes” [tiab] OR interleukin [tiab] OR “lipidemic profile” [tiab] OR “lipid profile” [tiab] OR “lipid levels” [tiab] OR “endothelial dysfunction” [tiab]
#4 intervention [tiab] OR trial [tiab] OR RCT [tiab] OR “randomized controlled trials” [tiab]
#1 AND #2 AND #3 AND #4

**Table 2:** Presentation of included studies.

Study Name	Publication Year	Journal	Origin	Ethical Permission	RCT Design	Randomization	Masking
Di Pino et al. [23]	2016	Journal of Clinical Lipidology	Italy	✓	Parallel, controlled	Computerized by random number generator	Blinded physician
Lotan et al. [24]	2020	Nutrients	Israel	✓	Parallel	✓	Blinded research staff, except the study dietitian
Steenbeke et al. [26]	2022	Nutrients	Belgium	✓	Parallel	✓	No
Cai et al. Vlassara et al. [22,28]	2002 2004	Circulation	USA	✓	Crossover and Parallel	✓	N/A
Luévano-Contreras et al. [25]	2013	Journal of Clinical Biochemistry and Nutrition	Mexico	✓	Parallel	Computerized by random number generator	Blinded physician
Uribarri et al. [27]	2011	Diabetes care	USA	✓	Parallel	✓	N/A

N/A = not available.

**Table 3:** Basic characteristics of included studies.

Study Name	Patients, n	Men, n	Age, y	Diabetes Criteria	Intervention, n	Control, n	Inclusion/Exclusion Criteria
Di Pino et al. [23]	62	N/A	54.8 ± 8.4	according to the ADA recommendation	L-dAGEs = 31	S-dAGEs = 31	Inclusion: Prediabetic patients; 35–65 y; 18.5–40 BMI; Caucasian race Exclusion: ≥190 mg/dL LDL levels at baseline; previous history of diabetes; previous history of CVDs; smokers; liver/renal disease; anemia/hemoglobinopathies; medications affecting glucose or lipid profile; use of vitamin supplements; major food allergies; history of eating disorders; eating habits different from MD; high weight loss or change in dietary habits the last 3 months; chronic GI associated with malabsorption or chronic pancreatitis; rheumatic diseases; history of acute illness, malignant disease, and drug or alcohol abuse
Lotan et al. [24]	75	56	71.6 ± 4.1	from medications for T2DM, health records or two recent routine blood tests (fasting glucose > 126 mg/dL).	35	40	Inclusion: >65 y, T2DM diagnosis and subjective memory complaints Exclusion: Any existing neurological condition that can affect cognition (e.g., dementia, Parkinson’s disease, schizophrenia, traumatic head injury, etc.)
Steenbeke et al. [26]	40 with Diabetic Nephropathy	29	73.9 ± 6.9	Not mentioned	20	20	N/A
Cai et al. Vlassara et al. [22,28]	24	11	L-dAGE = 62 ± 5 S-dAGE = 61.7 ± 7	Patients with know T2DM with good metabolic control	L-dAGEs = 13 American Diabetes	S-dAGE = 11	Exclusion: HbA1c > 9%, frequent hypoglycemia, cardiac event within the preceding 3 months, glucocorticoids or anticoagulants (other than aspirin), sCr ≥ 176.8 μmol/L, or had food allergy or religious restrictions
Luévano-Contreras et al. [25]	26	3	46 ± 5 (L-dAGEs) 48.5 ± 6.2 (S-dAGEs)	Not mentioned	L-dAGEs = 13	S-dAGEs = 13	Inclusion: 30–65 y with <10 y since T2DM diagnosis; sCr < 1.2 mg/dL; stable weight on the last 2 months; no food allergies and no clinical evidence of infections and serious condition (e.g., cancer, liver/heart disease) Exclusion: changes in the medication for diabetes or reported unsatisfactory adherence to diet
Uribarri et al. [27]	36	8	61 ± 4 (DMG) 67 ± 1.4 (CG)	Not mentioned	DMG = 18 (L-dAGEs = 12 S-dAGEs = 6)	CG = 18 (L-dAGEs = 9 S-dAGEs = 9)	Exclusion: renal disease or overt CVDs

y = years; N/A = not available; ADA = American Diabetes Association; L-dAGEs = low advanced glycation end-products diet; S-dAGEs = standard advanced glycation end-products diet; BMI = body mass index; LDL = low density lipoprotein; CVDs = cardiovascular diseases; MD = Mediterranean diet; GI = gastrointestinal diseases; T2DM = type 2 diabetes mellitus; etc. = et cetera; HbA1c = hemoglobin A1C; sCr = serum creatinine; DMG = diabetes mellitus group; CG = control group.

**Table 4:** Results of low-AGE diet (L-dAGEs) interventions.

Study Name	Intervention Details	Comparator Details	Duration	Timepoints of Measurements	Primary Outcomes	Secondary Outcomes	Results
Di Pino et al. [23]	L-dAGES: received written indications for food preparation, cooking time and temperature, were advised to avoid industrial food known to be cooked at high temperature and prefer minimally processed products	S-dAGES: advised to maintain their usual cooking habits.	24 weeks	Baseline: measurements of clinical, dietary and biochemical (fasting glycemia, lipid profile and hs-CRP) characteristics, receptors for AGEs, arterial stiffness and carotid atherosclerosis 12 weeks: same to baseline 24 weeks: fasting glycemia, lipid profile, hs-CRP, ApoB, ApoA, esRAGE and cardiovascular risk evaluation	the effect of L-dAGE on lipid profile, inflammatory markers, and plasma levels of HbA1c, TC, non-HDL, HDL, TGs, ApoB, ApoA, esRAGE, and hs-CRP	the effects of an L-dAGE regimen on early markers of CVDs	12 weeks ↓ TGs, TC, LDL, and non-HDL in L-dAGEs group compared to baseline and S-dAGEs group ↓ HDL in the L-dAGEs group compared to S-dAGEs ↓ TGs in the S-dAGEs compared to baseline No change in glucose 24 weeks ↓ TC, LDL, and non-HDL in L-dAGE group from baseline and between the groups ↓ ApoB and ApoB/ApoA ratio in L-dAGE group compared to baseline and S-dAGE group ↓ hs-CRP levels in L-dAGE group from baseline ↓ IMT in the L-dAGE group from baseline
Lotan et al. [24]	L-dAGEs group: dietary guidelines for good glycemic control, focused on reducing dietary AGEs intake	S-dAGEs group: dietary guidelines for achieving good glycemic control. Recommendations carbohydrate intake from various sources	6 months	Baseline: measurements of markers related to diabetes (glucose, HbA1c, insulin HOMA-IR), lipid profile (TGs, HDL, LDL, TC), kidney function (Urea, sCr), CRP, sAGEs (CML, CEL, G-H1, MG-H1, and 3DG-H1) 6 months: same to baseline	Change in sAGEs after the intervention	Diet association with sAGEs	↓ estimated dietary intake of AGEs in L-dAGEs group compared to S-dAGEs group ↓ in sAGEs levels, (CEL, 3DG-H1, and MG-H1) in participants classified as “very highly adherent” to the AGE-lowering diet No changes in glucose, HbA1c
Steenbeke et al. [26]	L-dAGEs group: provided information about dAGEs and the cooking methods to reduce the consumption of dAGEs	S-dAGEs group: Usual nutrition	8 weeks	Baseline: HbA1c, Serum and urinary Cr eGFR, sAGEs 8 weeks: same to baseline	Changes in measurements after 8 wks from baseline	-	No changes in HbA1c in and between the groups No changes in AGEs content between the two groups
Cai et al. Vlassara et al. [22,28]	L-dAGEs group: Two diets, differing in AGEs content were used both dietary groups received similar vitamin supplements (vitamins E & C)	S-dAGEs group: The difference in AGEs content of foods was achieved largely by modifying the cooking time and temperature	2 weeks crossover 6 weeks parallel	Baseline: LDL, glucose, HbA1c, fasting lipid profiles, sAGEs, and LDL-associated AGEs, TNF-α, mRNA, CRP 2 weeks: serum AGEs levels, TNF-α mRNA, CRP 6 weeks: same to baseline	Changes in measurements after 6 weeks from baseline		2 weeks’ crossover: ↑ sAGEs on S-dAGEs group and ↓ on L-dAGEs group During the crossover study, there were no significant differences in glucose control, TC and TG ↓ TNF-α mRNA in mononuclear cells on the L-dAGEs group compared to S-dAGEs group 6 weeks parallel: ↑ CRP and mononuclear TNF-α protein on S-dAGEs group and ↓ on L-dAGEs group ↓ Glucose on L-dAGEs group compared to S-dAGEs No significant differences between the baseline and 6 wks within each group in HbA1c, fasting plasma glucose and lipid profile ↑ in total sAGE levels on S-dAGEs group and ↓ on L-dAGEs group LDL from diabetic patients on S-dAGEs diet was significantly more glycated and oxidized compared with normal LDL LDL from diabetic patients on L-dAGE group was less glycated (by 50%) and less oxidized S-dAGEs group
Luévano-Contreras et al. [25]	L-dAGEs group: received written indications for food preparation, cooking time and temperature advised boil and steam the food, to avoid fried entrees and reheat food indirectly using steam in a double boiler.	S-dAGEs group: advised to maintain their usual cooking habits.	6 weeks	Baseline: Anthropometric measurements, serum glucose and lipids, HbA1c, CRP, TNF-α, MDA 6 weeks: same to baseline	The effect of dAGEs restriction on TNF-α, MDA, CRP, and insulin resistance	-	↓ of MDA and TNF-α in the L-dAGEs group compared to S-dAGEs group No changes in glucose, HbA1c, HOMA, total-cholesterol, HDL-cholesterol and triglycerides
Uribarri et al. [27]	DMG: received instructions on how to modify cooking time and temperature advised to boil, poach, stew, or steam food and to avoid frying, baking, or grilling, methods, to restrict AGEs	CG: same advices to DMG	4 months	Insulin leptin, Adiponectin, TNF-α, AGEs, 8-isoprostanes	Changes in measurements after 4 months from baseline	-	DM patients L-dAGEs group: ↓ sCML, sMG, iMG, Insulin, HOMA-IR, 8-isoprostanes from baseline ↑ AGER1, SIRT-1 in peripheral blood mononuclear cells, adiponectin from baseline S-dAGEs group: ↑ sMG, iCML, TNF-α, Leptin from baseline ↓ Adiponectin from baseline L-dAGEs vs. S-dAGEs groups: ↓ sCML, sMG, iCML, iMG, RAGE (mRNA), TNF-α, insulin, HOMA, Leptin and in S-dAGEs group these markers were increased in L-dAGEs vs. S-dAGEs AGER1, SIRT-1 in peripheral blood mononuclear cells, Adiponectin ↑ in L-dAGEs and ↓ in S-dAGEs groups and the differences were significant between the groups over time Healthy participants L-dAGEs group: ↓ sCML, sMG, and 8-Isoprostane from baseline L-dAGEs vs. S-dAGEs groups: ↓ sCML, sMG, RAGE (mRNA), TNF-α and 8-Isoprostane in L-dAGEs and ↑ in S-dAGEs and the differences were significant between the groups over the time

L-dAGEs = low advanced glycation end-products diet; S-dAGEs = standard advanced glycation end-products diet; hsCRP = high-sensitivity C-reactive protein; AGEs = advanced glycation end products; ApoB: Apolipoprotein B; ApoA: Apolipoprotein A; esRANGE = endogenous secretory receptor for AGEs; DM = diabetes mellitus; DMG = diabetes mellitus group; HbA1c = hemoglobin A1C; TC = total cholesterol; non-HDL: high-density lipoprotein; TGs = total triglycerides; CVDs = cardiovascular diseases; LDL = low-density lipoprotein; IMT: intima-media thickness; HOMA-IR: homeostatic model assessment of insulin resistance; sCr = serum creatinine; sAGEs = serum advanced glycation end products; CML = Ne-carboxymethyl lysine; CEL = Ne-carboxyethyl lysine; G-H1 = Glyoxalhydroimidazolone; MG-H1 = Methylglyoxalhydroimidazolone; 3DG-H1 = 3-deoxyglucosone hydroimidazolone; Cr = Cretinine; eGFR = glomerular filtration rate; TNF-α = tumor necrosis factor-α; mRNA = messenger ribonucleic acid; CRP: C- reactive protein; MDA: malondialdehyde; sCML = serum Nε-(1-Carboxymethyl)-L-lysine; sMG = serum methylglyoxal; iMG = intracellular methylglyoxal; AGER1 = advanced glycation end product receptor-1; SIRT-1 = silent mating-type information regulation 2 homolog 1; RAGE = receptor for AGEs; vs = vers.

**Table 5:** Risk of bias assessment of the included RCTs.

	Cai et al. [22]	Di Pino et al. [23]	Lotan et al. [24]	Luévano-Contreras et al. [25]	Steenbeke et al. [26]	Uribarri et al. [27]	Vlassara et al. [28]
S1	some concerns	Low Risk	some concerns	Low risk	some concerns	some concerns	some concerns
S2	some concerns	some concerns	some concerns	some concerns	some concerns	some concerns	some concerns
S3	some concerns	some concerns	some concerns	some concerns	some concerns	some concerns	some concerns
S4	Low Risk	Low Risk	some concerns	some concerns	Low risk	Low risk	Low risk
S5	some concerns	Low Risk	Low Risk	some concerns	some concerns	Low risk	some concerns
S6	some concerns	some concerns	some concerns	some concerns	some concerns	some concerns	some concerns

S1: random sequence generation. S2: deviation from the intended interventions. S3. measures of the outcomes. S4: incomplete outcome data. S5: selective reporting. S6: overall bias.

## Supplementary Materials

The following supporting information can be downloaded at: https://www.mdpi.com/article/10.3390/ijms252111407/s1.
